# Characterization of thrombosis risk in ambulatory patients with cancer: results of the observational, prospective, multicenter CARTAGO study

**DOI:** 10.1093/oncolo/oyae334

**Published:** 2024-12-02

**Authors:** Javier Trujillo-Santos, Ignacio García-Escobar, Mercedes Salgado, António Araújo, Eva Martínez-de-Castro, Raquel Molina, Victoria E Castellón-Rubio, Pere Domènech, Enrique Gallardo, Esteve Colomé, Ferran Torres, José-Carlos Benítez-Montañez, Rut Porta, Míriam Lobo-de-Mena, Mariana Malheiro, Carme Font, Elena Brozos-Vázquez, Fernando Garicano, Víctor Sapena, Ana-Lucia Costa, Ana-Cristina Albuquerque, Pablo Cerezuela, the CARTAGO Study Group, Sara Agraso, Georgia Anguera, Maite Antonio, David Arias, Mercedes Biosca, Ana Blasco, Anna Bustins, Diego Cacho, Núria Calvo, Begoña Campos-Balea, Marta Carmona, Elena Cillan, Carmen Díaz-Pedroche, Paula Espinosa Olarte, Francis Exposito, Isaura Fernández, Lourdes Fernández-Franco, Tomeu Fullana, Silvia García-Adrián, Javier García-Sánchez, Sandra Giménez, Irene González-Cebrián, Manuel González Moya, Clara Lucía Gozálvez, David Gutiérrez-Abad, Yolanda Lage, Rosa López, María Luisa Limón, Raquel Luque, Agustín Hernández, Evelin Horvath, Rosa López, Ismael Macias, Montse Mangas, Pascual Marco, Edelmira Martí, Purificación Martínez-del-Prado, Maria Masvidal, Marina Meri, Marta Merino, Yolanda de Miguel, Santiago Moragon, Daniela Morello, Berta Obispo, Montse Pàmpols, Pedro Pérez-Segura, Beatriz Rivas, Alberto Rodrigo, Silverio Ros, Araceli Sabino-Álvarez, Diego Salgado, Raúl Sánchez, Lucía Teijeira, María Valero-Arbizu, Francisca Vazquez, Jose Carlos Villa, Paula Alves, Júlia Amorim, Anabela Barros, Carolina Carvalho, Nuno Couto, Ana Rita Garcia, Vitória Gemas, Beatriz Gosalbez, Hélder Mansinho, Jorge Martinez, Mafalda Peres, António Moreira Pinto, Catarina Pulido, Ana Raimundo, Maria João Ribeiro

**Affiliations:** Internal Medicine Department, Santa Lucía General University Hospital, Catholic University of Murcia, 30202 Murcia, Spain; Medical Oncology Department, General University Hospital of Toledo, 45007 Toledo, Spain; Medical Oncology Department, Ourense University Hospital Complex, 32005 Ourense, Spain; Santo António University Hospital Centre, School of Medicine and Biomedical Sciences (ICBAS), University of Porto, 4099-001 Porto, Portugal; Medical Oncology Service, Marqués de Valdecilla University Hospital, Valdecilla Research Institute (IDIVAL), 39008 Santander, Spain; Medical Oncology Department, Príncipe de Asturias Teaching Hospital, 28805 Alcalá de Henares, Spain; Medical Oncology Department, Torrecárdenas University Hospital, 04009 Almería, Spain; Haematology Service, Bellvitge University Hospital, 08907 Barcelona, Spain; Medical Oncology Service, Parc Taulí University Hospital, Parc Taulí Research and Innovation Institute (I3PT-CERCA), Autonomous University of Barcelona, 08208 Sabadell, Spain; Medical Affairs Department, LEO Pharma, 08003 Barcelona, Spain; Biostatistics Unit, Medical School, Autonomous University of Barcelona, 08193 Barcelona, Spain; Medical Oncology Department, Mútua de Terrassa Hospital, 08221 Barcelona, Spain; Medical Oncology Department, Catalan Institute of Oncology (ICO), 17007 Girona, Spain; Medical Oncology Department, General University Hospital, 46014 Valencia, Spain; Medical Oncology, São Francisco Xavier Hospital, Lisboa Ocidental Hospital Centre (CHLO), 1449-005 Lisbon, Portugal; Medical Oncology Department, Clinic Hospital, 08036 Barcelona, Spain; Translational Medical Oncology Group, Oncomet, Santiago de Compostela University Hospital Complex (CHUS), 15706 Santiago de Compostela, Spain; Galdakao Hospital, 48960 Galdakao, Spain; Biostatistics Unit, Medical School, Autonomous University of Barcelona, 08193 Barcelona, Spain; Oncology Division, Santa Maria Hospital, Lisboa Norte Hospital Centre (CHLN), 1649-028 Lisbon, Portugal; Medical Oncology Department, Setúbal Hospital Centre, 2910-446 Setúbal, Portugal; Medical Oncology Department, Virgen de la Arrixaca Clinical University Hospital, 30120 Murcia, Spain

**Keywords:** D-dimer, leukocytes, thromboprophylaxis, venous thromboembolism, cancer

## Abstract

**Background:**

Venous thromboembolism (VTE) is one of the leading causes of death in patients with cancer. Currently, there is a need to develop an easily applicable risk model that can identify patients who will benefit from receiving primary thromboprophylaxis to reduce the incidence of VTE.

**Patients and methods:**

This was a non-interventional, multicenter, observational, prospective study carried out in 62 Oncology and Hematology services in Spain and Portugal between January 2018 and December 2019. The main objective of the CARTAGO study was to develop a predictive model within a competitive risk framework to assess the risk of VTE in patients with cancer undergoing chemotherapy, biological, or hormonal treatment.

**Results:**

A total of 1596 patients were analyzed. VTE events occurred in 124 (8%) during the 6-month follow-up period (42% of deep vein thrombosis [DVT], 48% of pulmonary embolism [PE], and 10% of both DVT and PE). Four variables were selected for the multivariate predictive model to determine the risk of VTE (tumor type, D-dimer, compression of a vessel by the tumor, and leukocyte count). The 4 variables were associated with an increased risk of VTE (C-statistic, 0.646 [95%CI, 0.620-0.673]). The most significant variables in the internal validation with bootstrapping were the “very high risk” tumors (hazard ratio [HR] 2.032; 95%CI, 1.287-3.211).

**Conclusion:**

The CARTAGO model predicts the VTE risk in patients with cancer receiving anticancer therapy in an outpatient setting. This model can easily aid in identifying ambulatory patients who would probably benefit from primary thromboprophylaxis.

**Implications for practice**

The occurrence of thrombotic events is increasing and negatively impacts the survival of cancer patients, with venous thromboembolism being one of the leading causes of death. Current scales used for the detection of patients at thromboembolic risk have a series of limitations. The CARTAGO model may predict the risk of developing venous thromboembolism in ambulatory cancer patients treated with anticancer therapies. It is based on 4 easily obtained parameters (tumor type, D-dimer, vessel compression by tumor, and leukocytes) and can help clinicians identify patients who should receive primary thromboprophylaxis.

## Introduction

Venous thromboembolism (VTE) represents a frequent complication in patients with cancer.^1^ The incidence of VTE associated with cancer is significantly elevated, especially during the first months after diagnosis.^2^ Its occurrence depends on various risk factors, such as the type of tumor or the anticancer treatment received, and it is increasing due to the improvement of imaging techniques for diagnosis, the increasing age of patients, as well as the greater capacity of new treatments to generate thrombotic events.^1,3^ It is possible that the occurrence of VTE often reflects the aggressiveness of the cancer itself.^4^ In addition, VTE can cause additional morbidity and affect cancer treatment, being a prognostic factor with a negative impact on survival.^5^ Therefore, a tool that can adequately define the risk of experiencing a thromboembolic event is needed to identify the patients with a favorable risk/benefit ratio who would receive thromboprophylaxis.^4-7^

Several scales have been established for the detection of patients at thromboembolic risk. The most widely used is the Khorana score, although it has a series of limitations, such as a short follow-up period, a low representation of certain tumors with a clear association with VTE and the fact that most VTE cases occur outside the high-risk patient group. The Vienna Cancer and Thrombosis Study (CATS) performed for the external validation of Khorana score included 2 new biomarkers, D-dimer and P-selectin, and was able to improve the VTE predictive capacity. However, its drawback is the lack of validation and the difficulty of routine determination of P-selectin.^8^ In the attempts to simplify the Vienna CATS score, a simpler model that focused only on tumor-site and D-dimer concentration was developed.^9^

There are 2 other validated risk scales, the ONKOTEV and the Tic-Onco genetic risk score, which, however; also present limitations when applied in clinical practice.^10,11^ The ONKOTEV scale includes: metastatic disease, macroscopic vascular, or lymphatic compression associated with malignancy, and a history of VTE.^10^ The Tic-Onco clinical-genetic risk score identifies patients at high risk of VTE, including 5 variables: tumor type, cancer stage, body mass index (BMI), family history, and the genetic profile of the patient.^11^

Most thromboembolic events in cancer occur in ambulatory patients receiving systemic anticancer treatment. However, primary thromboprophylaxis is not justified in all of these patients, and thus, the identification of patients at high risk is of particular interest in this setting.^6^ The CARTAGO study was mainly undertaken in outpatients suffering from digestive, pulmonary, non-Hodgkin lymphoma, genitourinary, and gynecologic cancers. The aim of the study was to identify the most relevant risk factors for VTE and to develop a predictive risk model applicable to patients initiating anticancer treatment.

## Patients and methods

### Study design

This was a non-interventional, multicenter, observational, and prospective study that aimed to develop a new predictive model of thrombotic events in oncology patients undergoing anticancer therapy. The study was performed in 62 Oncology and Hematology services in Spain and Portugal between January 2018 and December 2019.

This study was approved by the Ethics Committee of Hospital General Universitario Santa Lucía (Cartagena) in Spain and the Centro Hospitalar Universitário Santo António (Porto) in Portugal. Informed consent was obtained from each patient before any data could be recorded. The study was conducted in accordance with the requirements expressed in the international standards for the conduct of epidemiological studies.^12^

### Patient selection

The study included patients older than 18 years with different stages of cancer who were going to start cancer treatment. Patients who were planning to undergo surgery for cancer treatment were excluded, as well as those with tumors that had a VTE incidence rate lower than 10 events per thousand people per year.^13^ Therefore, patients with breast cancer treated with adjuvant therapy, metastatic breast cancer treated only with hormone therapy, and non-metastatic or metastatic hormone-sensitive prostate, head and neck, larynx, melanoma or thyroid cancer were not included in the study. In addition, patients with low-incidence tumors, such as esophageal cancer, mesothelioma, Kaposi’s sarcoma, bone tumors, cervical cancer, and Hodgkin’s lymphoma, were also excluded.^14^ An Eastern Cooperative Oncology Group (ECOG) performance status (PS) between 0 and 2 and a life expectancy of ≥12 weeks was also required for inclusion, as well as the absence of any psychological, sociological, or geographical situation that could hinder compliance with the study protocol and follow-up schedule.

Patients who had received palliative radiotherapy or major surgery within 14 days prior to inclusion in the study were excluded. They could not have any recent history of arterial or venous thrombosis. Patients were also excluded if they were under anticoagulant therapy at the time of inclusion in the study or if the physician expected to use ambulatory thromboprophylaxis. However, the patient was eligible if anticoagulants were required due to hospitalization for an acute process. Unwillingness or inability to comply with the study and its follow-up procedures was also an exclusion criterion.

### Study objectives and procedures

The main objective of this study was to develop a predictive model for the risk of VTE in patients with active cancer who were undergoing chemotherapy (CT), biological treatment, or hormonal treatment (except in the case of breast cancer). Secondary objectives were to determine the incidence and characteristics of thrombotic and hemorrhagic events in these patients.

Each investigator recorded the demographic and epidemiological data, vascular risk factors, tumor type, disease stage, treatments administered (chemotherapy, hormonotherapy, radiotherapy, biological therapy, colony-stimulating factors, etc.), potential inflammatory and thrombotic markers (lactate dehydrogenases [LDH], neutrophil-lymphocyte ratio [NLR], D-dimer, platelet-lymphocyte ratio, C-reactive protein [CRP]), chronic comorbidities, and functional status of the patient on an electronic case report form. The primary event followed was the appearance of VTE, symptomatic or incidental, objectively confirmed, including deep vein thrombosis (DVT), pulmonary embolism (PE), both DVT and PE, central venous catheter (CVC) thrombosis, upper limb vein thrombosis, or vein thrombosis of rare localization (ie, splanchnic or cerebral vein thrombosis). VTE had to be documented by at least one of the following methods: Echo-Doppler, computerized tomography, magnetic resonance imaging, angiography, or scintigraphy. Secondary events followed were any episode of arterial thrombosis, hemorrhagic events, or death. Data collection was consecutive in all patients who met the selection criteria.

After the baseline visit, 2 additional visits were performed during the study, one in the third month after recruitment, and the other in the sixth month. However, the exact date of the onset of the events between visits was also recorded. The study analyzed patient status (alive or deceased), evolution (development or not of a thrombotic event), use of anticoagulants after a thrombotic event, and setting of the oncologic process (stable disease or progression).

### Statistical analysis

Categorical variables are expressed as absolute and relative frequencies (percentages), and continuous variables as the mean and standard deviation or median and interquartile range (IQR), as appropriate. The following baseline variables were imputed using multiple imputation (MI) based on the fully conditional specification method^15^ with 200 imputations^16^ height, weight, BMI, blood group, creatinine, platelets, neutrophils, lymphocytes, leukocytes, hemoglobin, LDH, D-dimer, CVC, and previous thrombosis. The MI method makes reliable estimations under the missing at random assumption despite the high percentages of missing values for some variables (such as 45% for blood group and 33% for D-dimer) observed in this study.^17^

The variables of time to thrombosis and death were evaluated using survival techniques. The survival function was described by the Kaplan-Meier method, and group differences were assessed by the log-rank test. For the evaluation of thrombosis, death before thrombosis was considered a competing risk^18^ and survival function was described using the cumulative incidence function.

**Figure 1.**
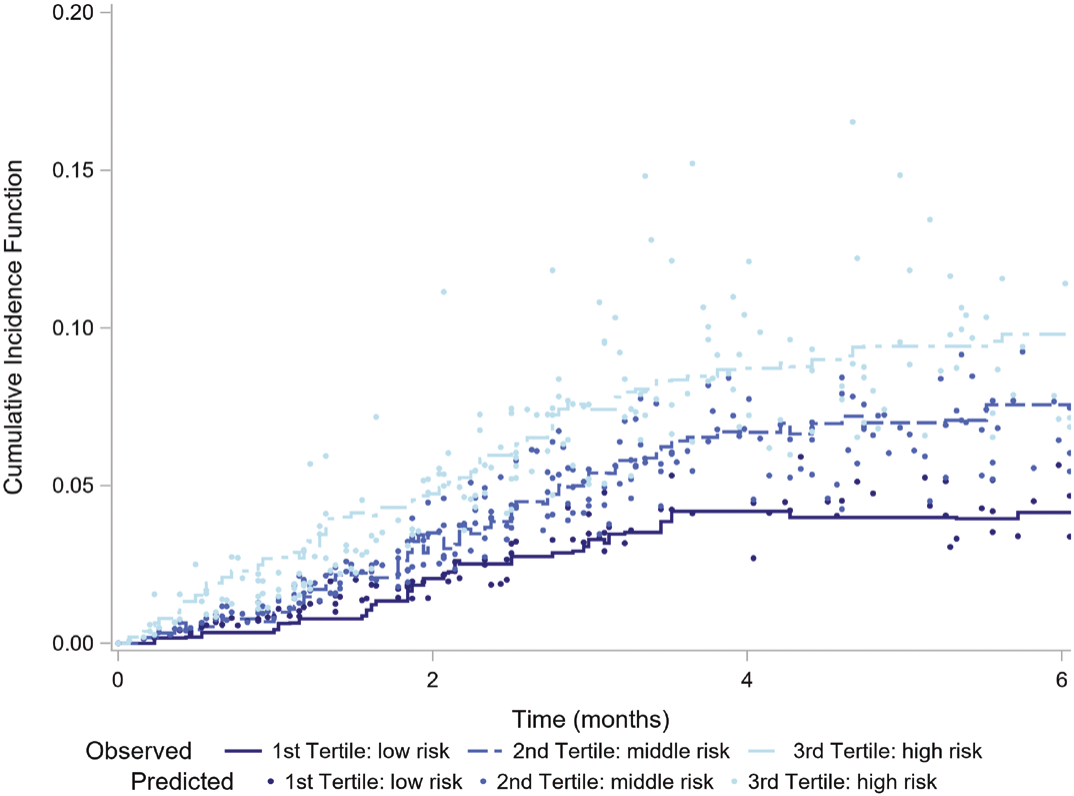
Survival plot with the model-predicted (markers) and observed (step lines) cumulative incidence function stratified by tertiles of predicted risk groups.

Cox regression models using subhazard distribution for handling competing events were used to select the best subset of predictors with assessed fit characteristics. Continuous variables were adjusted as continuous linear variables and with the use of quartiles, evaluating them as both categorical and ordinal variables with quartile change (trend test). Model selection was performed by combining clinical criteria with statistical criteria of discriminative utility using the *C*-statistic. Significance levels of 10% were adopted to introduce and eliminate variables from the model. All variables not selected for inclusion (*P* ≥ .10) were compared with the final model to determine whether their inclusion improved the model fit. Predicted probabilities from the Cox model were compared against the observed probabilities within a competing risk framework (Figure 1). The discriminative ability was assessed by Harrell’s *C*-statistic and its 95% CI as a criterion of the overall predictive model performance.^19^

The study followed the recommendations of the transparent reporting of a multivariable prediction model for individual prognosis or diagnosis (TRIPOD) statement.^20^ Given the lack of external data, an internal validation by bootstrap was conducted using 10 000 simulations. The analysis was performed using SAS software (SAS Institute Inc.) version 9.4, and significance was set at the 5% bilateral level.

## Results

### Patient characteristics at baseline

**Figure 2.**
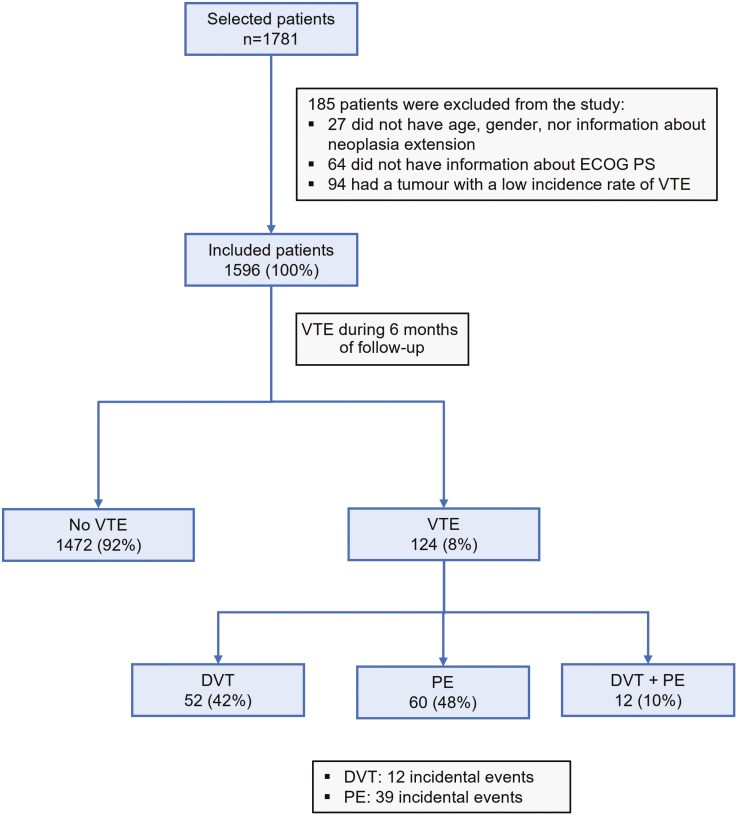
CONSORT diagram of the population of the CARTAGO study. CONSORT: Consolidated Standard of Reporting Trials. Abbreviations: DVT, deep vein thrombosis; ECOG PS, Eastern Cooperative Oncology Group performance status; PE, pulmonary embolism; VTE, venous thromboembolism.

Out of the 1781 cancer patients initially selected, 185 were excluded due to lack of basic information such as age, sex, tumor characteristics, ECOG PS, or because the incidence of tumors in our sample was very low. The baseline demographic and clinical characteristics of the 1596 patients finally included in this analysis are described in Figure 2 and Table 1.

**Table 1.** Distribution of baseline demographic characteristics of the study population according to the diagnosis of VTE.

Parameter	All cohort	No VTE	VTE
Patients			
Number (%)	1596 (100)	1472 (92)	124 (8)
Age (years), *n* = 1596			
Median [IQR]	67 [58–73]	67 [58–73]	67 [60–74]
Gender status, *n* = 1596			
Male, *n* (%)	1023 (64)	946 (92)	77 (8)
Female, *n* (%)	573 (36)	526 (92)	47 (8)
BMI (kg/m^2^), *n* = 1592			
Median [IQR]	25.4 [22.9–28.6]	25.3 [22.9–28.5]	26.0 [24–29.3]
ECOG PS, *n* = 1596			
0	723 (45)	677 (46)	46 (37)
1	748 (47)	683 (46)	65 (52)
≥2	125 (8)	112 (8)	13 (11)
Primary cancer site, *n* = 1596			
Colon and rectum	537 (34)	505 (34)	32 (26)
Non-small cell lung	374 (23)	341 (23)	33 (27)
Biliary and pancreatic	193 (12)	164 (11)	29 (23)
Stomach	155 (10)	145 (10)	10 (8)
Bladder and testicle	76 (5)	69 (5)	7 (6)
Small cell lung	69 (4)	67 (5)	2 (1)
Endometrial and ovarian	64 (4)	60 (4)	4 (3)
Lymphoma	51 (3)	45 (3)	6 (5)
Breast	49 (3)	48 (3)	1 (1)
Prostate	28 (2)	28 (2)	0 (0)
Tumour extension, *n* = 1596			
Localized	195 (12)	187 (13)	8 (6)
Locally advanced	526 (33)	487 (33)	39 (32)
Metastatic	875 (55)	798 (54)	77 (62)
Baseline laboratory values, median [IQR]			
Platelets (10^9^/L), *n* = 1585	266 [209–345]	267 [209–345]	260 [203–335.5]
Leukocytes (10^9^/L), *n* = 1592	7.7 [6.1–9.8]	7.7 [6.1–9.8]	8.2 [6.6–10.3]
Hemoglobin (g/dL), *n* = 1594	12.7 [11.3–14.0]	12.7 [11.3–14.0]	12.6 [11.4–13.9]
D-dimer (µg/L), *n* = 1076	758 [381–1550]	723 [370–1500]	1000 [532–2986]
CRP (mg/L), *n* = 1241	4.7 [1.1–17.3]	4.7 [1.1–17.0]	4.7 [1.1–27.3]
Anticancer treatment, *n* (%)			
Cisplatin	261 (16)	235 (16)	26 (21)
Gemcitabine	250 (16)	213 (15)	37 (30)
Irinotecan	78 (5)	65 (4)	13 (11)
Bevacizumab	98 (6)	93 (6)	5 (4)
Fluorouracil	383 (24)	352 (24)	31 (25)
Other, *n* (%)			
CVC	285 (18)	273 (96)	12 (4)
Vessel compression by adenopathy	82 (5)	70 (5)	12 (10)
Khorana score, *n* (%)			
0-1	901 (56.4)	839 (57.0)	62 (50.0)
2	453 (28.4)	413 (28.1)	40 (32.3)
≥3	242 (15.2)	220 (14.9)	22 (17.7)

Abbreviations: BMI, body mass index; CRP, C-reactive protein; CVC, central venous catheter; ECOG PS, Eastern Cooperative Oncology Group performance status; IQR, interquartile range; VTE, venous thromboembolism.

The median age of the patients was 67 years, and there was a higher proportion of men (64%). Among the patients included, 338 (21%) were active smokers, and the majority showed an ECOG PS of 0-1 (92%). Four types of cancer were of note among the study patients: colon and rectum (34%), non-small cell lung (23%), biliary and pancreatic (12%), and stomach (10%), and 55% of patients had metastatic disease. Most patients presented different comorbidities, such as hypertension (44%), hyperlipidemia (33%), diabetes mellitus (18%), chronic pulmonary disease (11%) or ischemic cardiopathy (7%). Sixty-eight patients (4.3%) had a history of any clinically relevant bleeding event (with 27 [1.7%] having a history of major bleeding events).

### Description of the incidence of thrombosis, bleeding, and mortality in the study population

VTE events occurred in 124 (8%) patients during the 6-month follow-up period, of which 52 (42%) were DVT, 60 (48%) were PE, and 12 (10%) were concomitant DVT and PE. Among the above, 12 incidental events were reported as DVT and 39 as PE (Figure 2). Eighty-two percent of VTE events were observed in the first 4 months.

The distribution of the baseline demographic characteristics of the population according to VTE occurrence is shown in Table 1 and the Supplementary Table S1. Regarding blood groups, VTE was less frequently observed in patients with blood type O (28% vs 41%). Of the 350 patients with blood group O, only 21 (6%) presented VTEs, whereas among 522 patients with other blood types, 53 (10%) developed VTE. The blood group was missing in 40% of the patients with VTE. The most thrombogenic tumor was biliopancreatic (29 VTE, 15%). The thromboembolic risk tended to be higher among tumors with metastatic involvement (VTE vs No VTE: 62% vs 54%) and lower in patients with local extension (6% vs 13%, respectively). Among baseline laboratory parameters, the median D-dimer value seemed to be higher in patients who exhibited VTE than in patients who did not (1000 µg/L vs 723 µg/L, respectively). Furthermore, the presence of vessel compression by adenopathy increased the incidence of VTE among study patients (VTE vs No VTE: 10% vs 5%).

Regarding anticancer treatment, patients who received gemcitabine seemed to be at higher risk of developing VTE (VTE vs No VTE: 30% vs 15%), followed by patients treated with irinotecan (11% vs 4%, respectively).

It was observed that there was a higher percentage of thromboembolic events among the 228 (14.3%) patients presenting tumor progression at 3 months: 29 (12.7%; *P* = .009). However, it was not a variable that could be selected at the beginning, and therefore could not be considered in the model.

In the study, 19 patients (1%) developed major bleeding, mainly gastrointestinal (84%). In 2 patients with an ECOG PS ≥3 at the 3-month follow-up visit, bleeding was fatal. Clinically relevant bleeding occurred in 2% of patients (17% occurred after the initiation of low-molecular-weight heparin [LMWH] to treat a VTE event). The tumors presenting a higher incidence of clinically relevant bleeding were gastric (7%), biliopancreatic (3%), non-small cell lung (3%), and colorectal (1%). Additionally, 340 patients (21%) died during the study period. Patients with metastases or with ECOG PS 2 had a higher mortality rate. Among patients who developed VTE during the study, mortality was also higher (36% vs 20% hazard ratio [HR]: 2.72; 95%CI, 1.96-3.77; *P* < 0.001). Considering mortality in patients with VTE, the tumors with the highest risk of death were biliopancreatic (VTE vs No VTE: 62% vs 31%), non-small cell lung (33% vs 27.5%) and stomach (40% vs 25%).

During follow-up, 20 (1.25%) arterial thromboses occurred (45% stroke, 35% myocardial infarction, and 20% peripheral arterial thromboses), with mortality in this group being 60%. Eighty percent of arterial events were observed in the first 3 months.

### Description of the univariate analysis

Univariate analysis with the imputed model of the baseline characteristics of the population presenting VTE is shown in Table 2 and Supplementary Table S2. Patients with an ECOG PS 0 tended to have a lower risk of VTE than patients with ECOG PS 2 (*P* = .134). The tumor with the highest risk of VTE was biliopancreatic (*P* < .001), followed by non-Hodgkin lymphoma (*P* = .067), and non-small cell lung cancer (*P* = .15). Additionally, a trend toward an increased risk of VTE was observed in patients with distant metastases compared with those without metastases (*P* = .053), showing a higher risk in patients with metastatic extension compared to those with localized disease (*P* = .027).

**Table 2.** Univariate analysis of the baseline demographic characteristics of the population presenting VTE, with missing data handled by multiple imputation.

	sHR (95% CI)	*P*-value variable	*P*-value for contrast
*Categorical variables*			
Gender			
Male (reference: female)	0.828 (0.570–1.202)	.321	
ECOG PS			
2	1 (Reference)	.134	
1	0.848 (0.459–1.567)		.598
0	0.599 (0.317–1.134)		.115
Primary cancer site			
Colorectal	1 (Reference)	<.001	
Biliopancreatic	2.914 (1.744–4.868)		<.001
Stomach	0.952 (0.436–2.079)		.902
Non-Hodgkin Lymphoma	2.304 (0.942–5.632)		0.067
Breast	0.37 (0.051–2.698)		.326
Ovary-endometrium	1.159 (0.409–3.288)		.781
Prostate	NE		NE
Non-small cell lung	1.452 (0.868–2.428)		.155
Small cell lung	0.529 (0.127–2.211)		.382
Testicle-bladder	1.754 (0.765–4.022)		.184
Tumour extension			
Localized	1 (Reference)	.063	
Locally advanced	1.888 (0.841- 4.238)		.124
Metastasis	2.391 (1.104–5.179)		.027
Compression of a blood vessel by the tumour adenopathy			
Yes (reference: no)	2.271 (1.243–4.149)	.007	
CVC			
Yes (reference: no)	0.906 (0.554–1.481)	.696	
Anticancer treatment (reference: no)			
Cisplatin	1.377 (0.876–2.160)	.164	
Gemcitabine	2.545 (1.718–3.759)	<.001	
Irinotecan	2.625 (1.475–4.673)	.001	
Bevacizumab	0.687 (0.281–1.684)	.412	
Fluorouracil	1.121 (0.740–1.701)	.590	
*Continuous variables and quartiles*			
Age at inclusion			
Age, per 1 year of age increase	1.007 (0.991–1.023)	.389	
BMI (kg/m^2^), Trend (per 1 quartile increase)	1.160 (0.991–1.357)	.064	
1st quartile	1 (Reference)	.280	
2nd quartile	1.387 (0.800–2.404)		.244
3rd quartile	1.238 (0.705–2.172)		.457
4th quartile	1.668 (0.982–2.834)		.058
Baseline laboratory values			
Platelets, per 1 × 10^9^/L increase	0.566 (0.101–3.170)	.517	
Leukocytes, per 1 × 10^9^/L increase	1.462 (1.080–1.979)	.014	
Hemoglobin, per 1 g/dL increase	0.981 (0.875–1.099)	.742	
D-dimer, per 1 µg/L increase	1.044 (1.001–1.089)	.043	
CRP, per 1 mg/L increase	1.032 (0.999–1.067)	.060	

Abbreviations: BMI, body mass index; CRP, C-reactive protein; CVC, central venous catheter; ECOG PS, Eastern Cooperative Oncology Group performance status; NE, not evaluable; sHR, subhazard ratio; VTE. venous thromboembolism.

The compression of a blood vessel by tumor adenopathy was clearly significant for VTE (*P* = .007). Among treatments given for cancer, gemcitabine was shown to have the most relevant effect on VTE risk (*P* < .001), followed by irinotecan (*P* = .001). Patients in the fourth quartile of the BMI showed a trend toward a higher risk of VTE compared to patients in the first quartile (*P* = .058). A relationship was observed between an increased leukocyte count and VTE (*P* = .014) and patients with VTE had an increased D-dimer level (*P* = .044). It is worth highlighting that among patients without VTE, those with metastasis had a median (IQR) D-dimer value higher than those without metastasis (876 [477-2069] vs 597 [305-1123]).

### Proposed VTE predictive model and internal validation with bootstrapping

All variables showing a *P* ≤ .20 were initially selected, including the blood group, ECOG PS, type of tumor, tumor extension, compression of a blood vessel by tumor adenopathy, anticancer treatment (irinotecan, gemcitabine, or cisplatin), leukocytes, and D-dimer. As shown in Table 3, 4 variables were selected for the multivariate predictive model to determine the risk of VTE. These variables included tumor type, D-dimer, compression of a vessel by the tumor and leukocytes. The effect of the tumor type was classified according to the Khorana score as low, high, and very high.

**Table 3.** Proposed VTE predictive model and internal validation with bootstrapping, with missing data handled by multiple imputation.

Variable	Model developed		Internal bootstrap validation
	sHR (95% CI)	p-value	sHR (95% CI)
Tumour type by Khorana^a^			
Low	1 (reference)	0.010	1 (reference)
High	1.614 (1.028-2.535)		1.647 (1.044-2.589)
Very high	2.066 (1.286-3.320)		2.041 (1.270-3.281)
D-dimer (quartiles)			
Per 1 quartile increase	1.191 (0.989-1.434)	0.065	1.244 (1.052-1.470)
Compression of a blood vessel by the tumor/adenopathy			
Yes (reference: absence)	1.908 (1.040-3.500)	0.037	1.893 (1.027-3.487)
Leukocytes			
Per 1 × 10^9^/L unit increase	1.029 (0.997-1.063)	0.079	1.028 (0.995-1.061)

^a^Very high-risk tumor type includes biliopancreatic and gastric cancer. High-risk tumor type includes lung, lymphoma, gynaecologic, bladder, and testicular cancer. Low-risk category includes other tumors.

C-statistic (95% CI): 0.642 (0.613-0.672); validation (95% CI): 0.649 (0.620-0.678).Abbreviations: sHR, subhazard ratio; VTE, venous thromboembolism.

The performance of the proposed predictive risk model was tested by evaluating its calibration and discrimination capacity. Internal validation was performed with bootstrapping, and the results were fully superimposable on the model built. Validation with bootstrapping was measured at a 95% CI. All HRs showed that the 4 variables were associated with an increased risk of VTE with a *C*-statistic of 0.648 (0.622-0.674) and partial area under the curve (AUC) at 1, 3, and 6 months of 0.648, 0.609, and 0.628, respectively. The most significant variables in the internal validation with bootstrapping were the very high risk in the Khorana tumor classification (HR 2.041; 95%CI, 1.270-3.281) and the compression of a blood vessel by tumor adenopathy (HR 1.893; 95%CI 1.027-3.487), which were independent predictive factors of VTE within 6 months as confirmed by the *C*-statistic of 0.642 (0.613-0.672) for the study and 0.649 (0.620-0.678) for the validation. Figure 2 presents the model-predicted and observed cumulative incidence function for the tertiles of predicted risk groups in the proposed predictive risk model, which showed a good discriminatory performance. The original Khorana and ONKOTEV scores demonstrated a *C*-statistic of 0.597 (95%CI, 0.568-0.627) and 0.626 (95%CI, 0.599-0.653), respectively. Supplementary Figures S1 and S2 showed the observed and predicted incidence values for Khorana and ONKOTEV scores. When comparing the performance of the selected model to the original Khorana and the ONKOTEV scores, the net reclassification index (95%CI) was 0.335 (0.159-0.591) with a *P*-value < .001 and 0.273 (0.088-0.457) with a *P*-value of .004, favoring the former. Other multivariate predictive models to determine the risk of VTE are reported in Supplementary Tables S3-S5. The coefficients needed to calculate the risk, along with an example of how to apply these coefficients, have been included in Supplementary Tables S6 and S7.

## Discussion

Anticancer treatments administered on an outpatient basis are an advantage for the patient. However, systemic CT is not without complications, including the development of VTE.^21^ Therefore, there is a need to select which ambulatory cancer patients are the most adequate candidates to receive primary thromboprophylactic therapy.

In agreement with the literature, in our study, 62% of VTE events occurred in stage IV patients.^22^ Although advanced age is assumed to be a thrombotic risk factor due to hemostatic and endothelial alterations leading to a hypercoagulable state,^23,24^ in our study, age was not found to have any influence on thrombotic risk. No association with other comorbidities, such as hypertension, hyperlipidemia, or diabetes, was observed. VTE occurred mainly (81% of patients) within the first 3 months after the onset of CT. This confirms that thrombotic risk is greater in the initial period after the diagnosis of cancer, possibly related to the onset of CT, which may contribute to hemostatic imbalance.^1^ In this study, an increase in VTE was observed not only when gemcitabine was administered but also with irinotecan treatment. However, the assessment of the role of these treatments is complex because the type and other characteristics of the tumor in which they were administered could have played a determinant role. Among 124 patients developing VTE, 51 cases were incidental events, which is consistent with other studies reporting that half of venous events in patients with cancer are discovered incidentally.^25^

Inflammation is one of the pathophysiological keys of VTE. Whether the relationship with increased inflammation is causal or a result of VTE can be debated. Our study included C-reactive protein, D-dimer, NLR, and platelet-to-lymphocyte ratio (PLR) as biomarkers of inflammation that are easily calculated from routine blood tests, with only D-dimer showing some relationship with VTE.

The CARTAGO predictive model included 4 easily identifiable variables, such as tumor type classified according to the Khorana score, D-dimer (quartiles), vessel compression by adenopathy, and leukocytes. It is important to highlight that the magnitude of the effect associated with D-dimer, especially at levels above 1550 ng/mL, may increase the risk of VTE 2-fold. Although the association of D-dimer with hypercoagulability has been considered for years, it should not be overlooked that D-dimer is an acute phase reactant, especially in patients with cancer. Increased D-dimer levels do not necessarily imply an increase in thrombotic activity and, thus, an increased risk of VTE.

The increase in leukocyte levels is also included in the Khorana score. However, unlike the Khorana score, with the CARTAGO model a relationship was observed with an increase of 1 × 10^9^/L units. A clear increase in thrombotic risk was also evident in the last quartile of this variable. In contrast, no relationship was observed between VTE and anemia or thrombocytosis.

Currently, none of the existing predictive models have reached an international consensus to accurately identify patients with cancer at greater risk of VTE associated with CT. Patient heterogeneity makes comparisons among studies difficult, sometimes leading to confounding variables.^26,27^ D-dimer is assessed in the Vienna CATS and the Multinational Cohort Study to Identify Cancer Patients at High Risk of Venous Thromboembolism (CATS-MICA) scales, which is of great importance as it only considers the tumor location apart from D-dimer values. The Vienna-CATS and Thrombo-NSCLC scales determine serum P selectin, which was not determined in the CARTAGO study as it is not used in routine clinical practice.^28^ The same applies for factor VIII assessed in the Thrombo-NSCLC scale. In our study, patients with a Khorana score ≥3 had a higher risk of VTE than those with a Khorana score <3. However, patients with a Khorana score <2 exhibited 6% of thrombotic events, and represented 50% of patients with VTE. This poor performance of the Khorana score has been observed in a meta-analysis including 3000 patients.^29^ The unsatisfactory results obtained with the Khorana score have prompted attempts to improve it by adding new variables. The Comparison of Methods for Thromboembolic Risk Assessment with Clinical Perceptions and AwareneSS in Real Life Patients-Cancer Associated Thrombosis (COMPASS-CAT) only agrees with Khorana when considering platelet count. In contrast, COMPASS-CAT includes variables such as tumor stage, cardiovascular risk factors, time since diagnosis and history of VTE. In contrast, the COMPASS-CAT included a heterogeneous sample of tumors and obtained adequate results with an AUC of 0.85.^30^ Subsequently, a second study reported good sensitivity but very low specificity. The ONKOTEV scale indicated a Khorana score of≥2 and considered the tumor stage, history of VTE, and vascular compression by the tumor. The authors demonstrated its superiority over the Khorana scale in a series of 843 patients with various types of tumors.^10^ An ONKOTEV score ≥2 was subsequently found to be associated with a higher rate of VTE in a retrospective study in pancreatic cancer.^31^ In our study, patients with an ONKOTEV score ≥1 had a higher risk of VTE than those with an ONKOTEV score of 0. However, patients with an ONKOTEV score of 0 exhibited 5.1% of thrombosis events, and with score 1 upwards, no increased risk of VTE was observed as the ONKOTEV score increased (Supplementary Appendix).

When comparing the 3 methods it can be concluded that, while the observed and predicted values for ONKOTEV and Khorana are closer than those of our proposed model, these 2 scores cannot adequately discriminate between risk levels in patients before 3 months, when the majority of events occur. Conversely, we acknowledge that in some cases, our proposed model might overestimate the risk of some high-risk patients. However, these patients are correctly assigned to the high-risk profile, and in general, there is minimal overlap in the incidence observed between the stratified risk levels.

In agreement with the literature, in our study, 62% of VTE events occurred in stage IV patients. Although the presence of distant metastasis showed a trend toward statistical significance (crude HR 0.729; 95%CI, 0.508-1.047; *P* = .0867), it was excluded from the final model because the prediction capacity of the model was lower and the statistical significance of the rest of the variables worsened. It is possible that the higher D-dimer values presented by patients with metastasis could be an interaction factor when both variables are included in the model (Supplementary Appendix).

Since all the variables considered by the CARTAGO predictive model are easily obtained in routine clinical practice, it would be very simple to apply. However, it would be interesting to increase the sample size and thereby facilitate the extrapolation of data to a more heterogeneous population. The fact that certain tumors were excluded from the study because they had a low incidence of VTE makes it difficult to extrapolate this model to all types of tumors. Additionally, the CARTAGO model has not been externally validated until now. Although an internal bootstrap validation was carried out, external validation with independent data is needed before the scale is widely used in routine clinical practice. Another limitation arises from missing data on some variables (such as 45% for blood group and 33% for D-dimer) that would have led to the exclusion of a substantial proportion of the original sample, which, in turn, would have caused a substantial loss of power. However, when we restricted the univariate analysis of these variables to individuals with complete information, there was a clear association between non-O blood type or D-dimer and VTE, and data imputation caused the loss of statistical significance of the blood group due to the high percentage of missing data.

## Conclusion

The CARTAGO model predicts VTE risk in patients with cancer receiving anticancer drugs in an outpatient setting. It is based on 5 parameters easily obtained in routine clinical practice, allowing clinicians to easily identify ambulatory patients who should receive primary thromboprophylaxis. We believe that the CARTAGO model may be very useful for the management of ambulatory cancer patients as well as for designing future trials involving outpatients with cancer. Nonetheless, an independent validation of the CARTAGO model should be performed.

## Supplementary Material

oyae334_suppl_Supplementary_Tables_S1-S7

oyae334_suppl_Supplementary_Figures_S1

oyae334_suppl_Supplementary_Figures_S2

## Data Availability

The data underlying this article will be shared on reasonable request to the corresponding author.
