# Supplementary material for: Characterization of thrombosis risk in ambulatory patients with cancer: results of the observational, prospective, multicenter CARTAGO study
Source: Oncologist. 2024 Dec 2;30(8):oyae334. doi: 10.1093/oncolo/oyae334 (PMC12395237; doi:10.1093/oncolo/oyae334)
Supplement: oyae334_suppl_Supplementary_Tables_S1-S7 [file oyae334_suppl_supplementary_tables_s1-s7.docx]

**Supplementary Appendix**

**Table S1.** Distribution of baseline demographic characteristics of the study population according to the diagnosis of VTE.

| **Parameter** | **All Cohort** | **No VTE** | **VTE** |
| --- | --- | --- | --- |
| **Blood group, n=872** |  |  |  |
| A, n (%) | 409 (47) | 366 (46) | 43 (58) |
| B, n (%) | 83 (10) | 75 (9) | 8 (11) |
| AB, n (%) | 30 (3) | 28 (4) | 2 (3) |
| O, n (%) | 350 (40) | 329 (41) | 21 (28) |
| **Baseline laboratory values, median [IQR]** |  |  |  |
| Neutrophil to lymphocyte ratio, n=1578 | 2.8 [1.9 - 4.5] | 2.8 [1.8 - 4.5] | 3.2 [2.1 - 4.9] |
| Platelet to lymphocyte ratio, n=1569 | 158 [113.3 - 235] | 158 [113.6 - 238.3] | 152 [107 - 215] |
| **Other, n (%)** |  |  |  |
| Previous VTE | 7 (~0) | 6 (~0) | 1 (1) |
| CVC | 285 (18) | 273 (96) | 12 (4) |
| CVC with PICC | 68 (24) | 68 (93) | 5 (7) |
| **ONKOTEV score, n (%)** |  |  |  |
| 0 | 598 (37.5) | 559 (38.0) | 39 (31.5) |
| 1 | 772 (48.4) | 709 (48.2) | 63 (50.8) |
| ≥2 | 226 (14.1) | 204 (13.8) | 22 (17.7) |
| **COMPASS-CAT score, n (%)** |  |  |  |
| Moderate risk: 0-6 | 453 (28.4) | 423 (28.7) | 30 (24.2) |
| High risk: ≥7 | 1143 (71.6) | 1049 (71.3) | 94 (75.8) |

VTE: Venous thromboembolism; CVC: central venous catheter; IQR: interquartile range; PICC: peripherally inserted central catheter.

**Table S2.** Univariate analysis of the baseline demographic characteristics of the population presenting VTE, with missing data handled by multiple imputation.

|  | **sHR (95% CI)** | **p-value**  **variable** | **p-value**  **for contrast** |
| --- | --- | --- | --- |
| **Categorical variables** |  |  |  |
| Blood group |  |  |  |
| O | 1 (Reference) | 0.440 |  |
| A | 1.418 (0.872 - 2.304) |  | 0.159 |
| AB | 0.967 (0.278 - 3.36) |  | 0.958 |
| B | 1.353 (0.638 - 2.865) |  | 0.430 |
| Not O group (Reference: O group) | 1.373 (0.863 - 2.184) | 0.180 |  |
| Distant metastasis |  |  |  |
| Yes (Reference: No) | 1.454 (0.995 -2.124) | 0.053 |  |
| History of venous thrombosis |  |  |  |
| Yes (Reference: No) | 1.958 (0.306 - 12.549) | 0.478 |  |
| BMI (kg/m^2^) |  |  |  |
| 1^st^ quartile | 1 (Reference) | 0.280 |  |
| 2^nd^ quartile | 1.387 (0.800 - 2.404) |  | 0.244 |
| 3^rd^ quartile | 1.238 (0.705 - 2.172) |  | 0.457 |
| 4^th^ quartile | 1.668 (0.982 - 2.834) |  | 0.058 |
| Baseline laboratory values |  |  |  |
| D-dimer |  |  |  |
| 1^st^ quartile | 1 (Reference) | 0.106 |  |
| 2^nd^ quartile | 1.492 (0.808 - 2.756) |  | 0.201 |
| 3^rd^ quartile | 1.437 (0.760 - 2.720) |  | 0.265 |
| 4^th^ quartile | 2.098 (1.159 - 3.799) |  | 0.014 |
| NLR, per 1 unit increase | 1.013 (0.981 - 1.046) | 0.430 |  |
| PLR, per 1 unit increase | 0.990 (0.972 - 1.008) | 0.278 |  |
| LDH, per 100 IU/L increase | 1.010 (0.714 - 1.429) | 0.955 |  |
| Khorana, per 1 unit increase | 1.128 (0.978 - 1.300) | 0.0971 |  |

BMI: body mass index; CI: confidence interval; CRP: C-reactive protein; CVC: central venous catheter; ECOG PS: Eastern Cooperative Oncology Group performance status; LDH: lactate dehydrogenases; NLR: neutrophil-lymphocyte ratio; PLR: platelet-lymphocyte ratio; sHR: subhazard ratio; VTE: venous thromboembolism.

**Table S3.** Proposed VTE predictive model with metastasis, including D-dimer.

| **Variable** |  | |
| --- | --- | --- |
|  | **sHR (95% CI)** | **p-value** |
| **Tumour type by Khorana^a^** | | |
| Low | 1 (Reference) | 0.010 |
| High | 1.612 (1.028 - 2.529) |  |
| Very high | 2.064 (1.286 - 3.314) |  |
| **D-dimer (Reference: 1st to 3rd quartiles)** | | |
| 4th quartile (Reference: 1st to 3rd quartiles) | 1.174 (0.972 - 1.418 | 0.095 |
| **Compression of a blood vessel by the tumour/adenopathy** | | |
| Yes (Reference: Absence) | 1.881 (1.028 - 3.439) | 0.04 |
| **Leukocytes** | | |
| Per 1x10^9^/L unit increase | 1.028 (0.993 - 1.063) | 0.118 |
| **Distant metastasis** | | |
| Yes (Reference: Absence) | 1.301 (0.88 - 1.921) | 0.187 |

C-statistic (95% CI): 0.665 (0.636-0.693).

CI: confidence interval; sHR: subhazard ratio; VTE: venous thromboembolism.

^a^Very high-risk tumour type includes biliopancreatic and gastric cancer. High-risk tumour type includes lung, lymphoma, gynaecologic, bladder and testicular cancer. Low-risk category includes other tumours.

**Table S4.** Proposed VTE predictive model with metastasis, excluding D-dimer.

| **Variable** | **Model developed** | | **Internal Bootstrap validation** |
| --- | --- | --- | --- |
|  | **sHR (95% CI)** | **p-value** | **sHR (95% CI)** |
| **Tumour type by Khorana^a^** | | | |
| Low | 1 (reference) | 0.010 | 1 (reference) |
| High | 1.606 (1.024 - 2.521) |  | 1.627 (1.033 - 2.563) |
| Very high | 2.064 (1.286 - 3.314) |  | 2.187 (1.362 - 3.510) |
| **Distant metastasis (yes)** | | | |
|  | 1.359 (0.925 - 1.995) | 0.059 | 1.369 (0.930 - 2.011) |
| **Compression of a blood vessel by the tumour/adenopathy** | | | |
| Yes (Reference: Absence) | 1.889 (1.037 - 3.444) | 0.019 | 1.867 (1.018 - 3.434) |
| **Leukocytes** | | | |
| Per 1x10^9^/L unit increase | 1.030 (0.996 - 1.066) | 0.092 | 1.030 (0.995 - 1.065) |

C-statistic (95% CI): 0.652 (0.624-0.681).

CI: confidence interval; sHR: subhazard ratio; VTE: venous thromboembolism.

^a^Very high-risk tumour type includes biliopancreatic and gastric cancer. High-risk tumour type includes lung, lymphoma, gynaecologic, bladder and testicular cancer. Low-risk category includes other tumours.

**Table S5.** Proposed VTE predictive model including chemotherapeutic drugs.

| **Variable** | **sHR (95%CI)** | **p-value** |
| --- | --- | --- |
| **Tumour type by Khorana^a^** |  |  |
| Low  High    Very High | 1 (Reference) | 0.0768 |
|  | 1.694 (1.069 – 2.685) |  |
|  | 1.499 (0.904 – 2.487) |  |
| **D-dimer (Reference: 1st to 3rd quartiles)** |  |  |
| 4th quartile (Reference: 1st to 3rd quartiles) | 1.454 (0.940 - 2.249) | 0.0925 |
| **Gemcitabine** |  |  |
| Yes (Reference: no exposure) | 2.194 (1.414 - 3.402) | 0.0005 |
| **Irinotecan** |  |  |
| Yes (Reference: no exposure) | 3.388 (1.821 - 6.301) | 0.0001 |

C-statistic (95%CI): 63.0 (60.3-65.7)

CI: confidence interval; sHR: subhazard ratio; VTE: venous thromboembolism.

^a^Very high-risk tumour type includes biliopancreatic and gastric cancer. High-risk tumour type includes lung, lymphoma, gynaecologic, bladder and testicular cancer. Low-risk category includes other tumours.

**Table S6.** Coefficients to be used for estimation of the linear predictor for a given set of covariates according to the predictive model in Table 3.

| Variable | Category/Value | Model coefficients for all cases | Model coefficients for  the reference | Example | |
| --- | --- | --- | --- | --- | --- |
|  |  |  |  | **Category/Values** | **Coefficient** |
| Tumour type by Khorana | Low | 0 | 0 | Very High | 0.725585788 |
|  | High | 0.478668479 |  |  |  |
|  | Very High | 0.725585788 |  |  |  |
| D-dimer (quartiles) | 1^st^ (coded as 0) | 0 | 0.2622076 | 4^th^ quartile | 0.524415193 |
|  | 2^nd^ (coded as 1) | 0.174805064 |  |  |  |
|  | 3^rd^ (coded as 2) | 0.349610129 |  |  |  |
|  | 4^th^ (coded as 3) | 0.524415193 |  |  |  |
| Compression of a blood vessel by the tumour adenopathy | No | 0 | 0 | Yes | 0.645830655 |
|  | Yes | 0.645830655 |  |  |  |
| Leukocytes (10^9^/L) (mean=8.5116) | Value | 0.028945915 | 8.5116*  0.028945915  =  0.2463789 | 15*10^9^/L | 15*  0.028945915  =  0.434188719 |
| Linear Predictor |  |  | $\boldsymbol{X}\boldsymbol{\beta}_{\boldsymbol{0}}$**=**  **0.5085865** |  | $\boldsymbol{X}\boldsymbol{\beta}_{\boldsymbol{1}}$**=**  **2.330020355** |

Example: [Tumour type by Khorana]=”Very High”, [D-dimer]=”4th quartile”, [Compression of a blood vessel by the tumour adenopathy]=”Yes” and [Leukocytes]=”15*10^9^/L”.

**Table S7.** Baseline survival $\hat{S}_{0}\left( t \right)$ at different cut-off time (t) periods in months according to the reference categories from Table S3.

|  | **1** | **2** | **3** | **4** | **5** | **6** |
| --- | --- | --- | --- | --- | --- | --- |
| $\hat{S}_{0}\left( t \right)$ | 99.21% | 97.93% | 96.59% | 95.94% | 95.69% | 95.49% |

For the running example, the calculated probability of the occurrence of a venous thrombosis event at 6 months would be:

$\hat{E}_{t}{=1-\hat{S}}_{t}=1-\left[ 0.9549 \right]^{\exp\left( \mathbf{2.330020355}-\mathbf{0.5085865} \right)}=0.2482\to24.82\%$.

**Figure S1.** Survival plot with the observed (step lines) and predicted (markers) incidence values for the Khorana cumulative incidence function stratified by tertiles of predicted risk groups.

**Figure S2.** Survival plot with the observed (step lines) and predicted (markers) incidence values for the ONKOTEV cumulative incidence function stratified by tertiles of predicted risk groups.
