# Supplementary figures and images for: Characterization of thrombosis risk in ambulatory patients with cancer: results of the observational, prospective, multicenter CARTAGO study
Source: Oncologist. 2024 Dec 2;30(8):oyae334. doi: 10.1093/oncolo/oyae334 (PMC12395237; doi:10.1093/oncolo/oyae334)

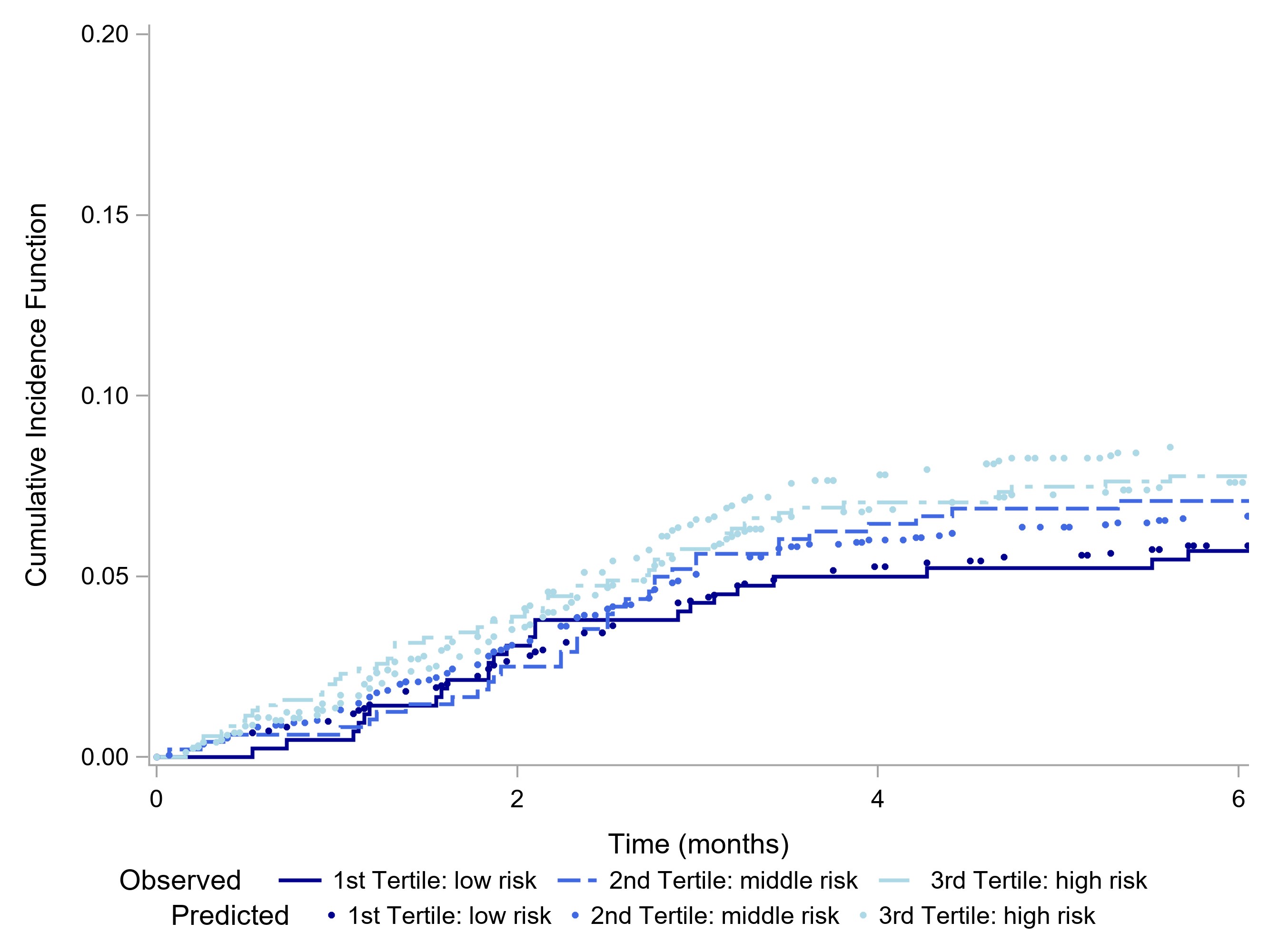

Supplement: oyae334_suppl_Supplementary_Figures_S1 [file oyae334_suppl_supplementary_figures_s1.jpeg]

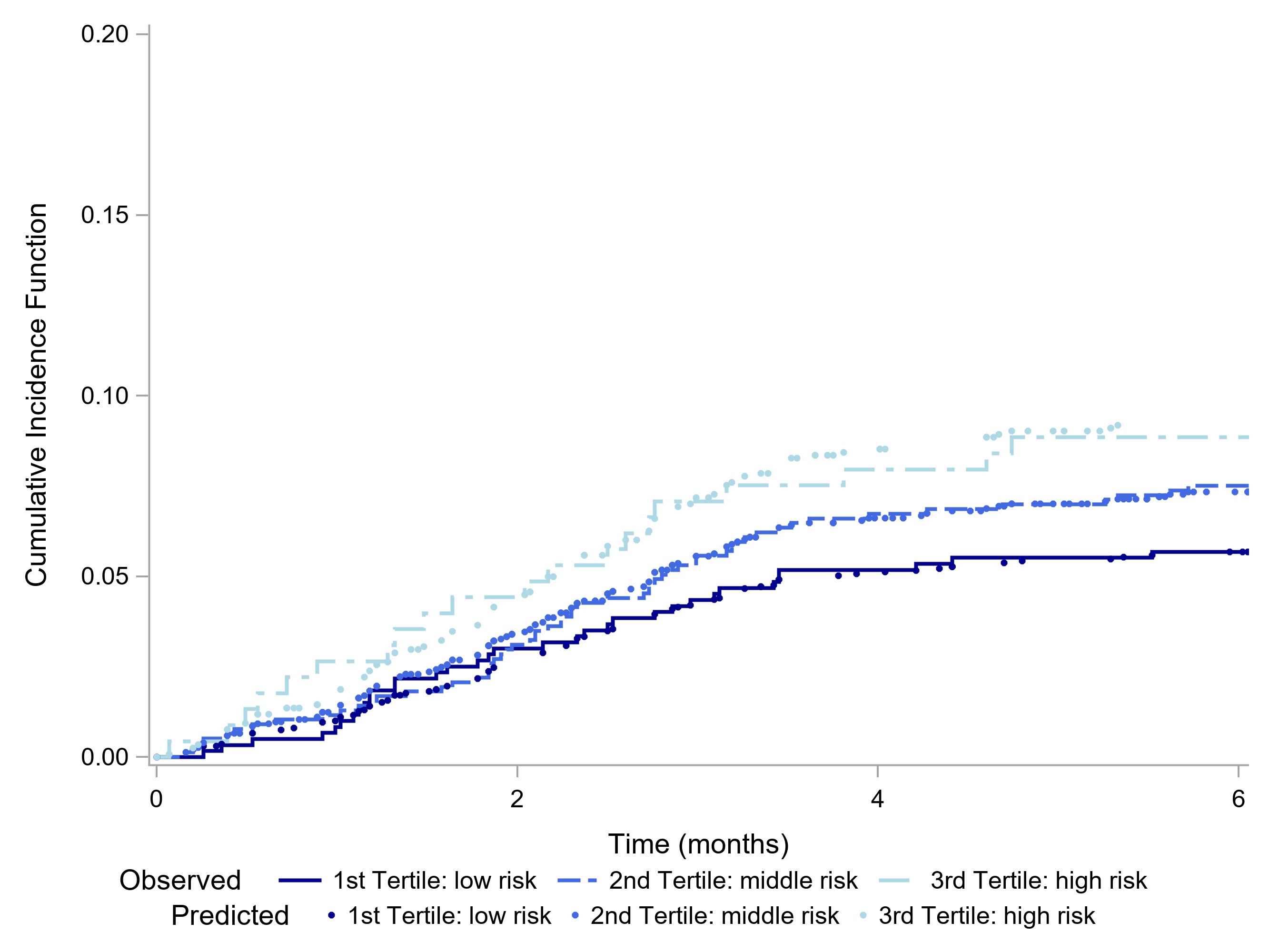

Supplement: oyae334_suppl_Supplementary_Figures_S2 [file oyae334_suppl_supplementary_figures_s2.jpeg]
